# Remodelling of the bone marrow microenvironment by stromal hyaluronan modulates the malignancy of breast cancer cells

**DOI:** 10.1186/s12964-020-00592-z

**Published:** 2020-06-09

**Authors:** Xiaoyan Chen, Xiaoxing Shi, Yiwen Liu, Yiqing He, Yan Du, Guoliang Zhang, Cuixia Yang, Feng Gao

**Affiliations:** 1 Department of Molecular Biology, Shanghai Jiao Tong University Affiliated Sixth People’s Hospital, Shanghai, 200233 People’s Republic of China; 2 College of Medical Technology, Shanghai University of Medicine and Health Sciences, Shanghai, 201318 People’s Republic of China; 3 Department of Laboratory Medicine, Shanghai Wujing General Hospital, Shanghai, 201103 People’s Republic of China; 4 Department of Clinical Laboratory, Shanghai Jiao Tong University Affiliated Sixth People’s Hospital, Shanghai, 200233 People’s Republic of China

**Keywords:** Hyaluronan, Mammary tumour, Bone metastasis, Bone marrow stromal cells

## Abstract

**Background:**

Hyaluronan (HA) is an abundant component of the bone marrow (BM) extracellular matrix. Here, we investigated the abnormal deposition of HA in the BM microenvironment and its remodelling in mediating the malignancy of breast cancer cells (BCCs).

**Methods:**

BCCs were transplanted into nude mice by intracardiac injection. The BCCs were cocultured with BM-derived stromal HS5 cells. Then, the abnormal metabolism of HA and its correlation with the malignant growth and the intracellular signalling pathways of the BCCs were investigated. After knockdown/out of the HA receptor CD44 in cancer cells by shRNA and CRISPR/Cas9, the mechanism was investigated in vivo through intratibial inoculation and in vitro by coculture with HS5 cells.

**Results:**

The malignancy of cancer cells was highly related to the degree of accumulation of HA in the BM. Further, stromal cell-derived HA, especially the mixed complex, significantly promoted the growth of BCCs and osteolysis by binding to the CD44 receptor. Additionally, the investigation of the underlying mechanism revealed that the PI3K, Cyclin D1, and CDK4 pathways were involved in the effect of bone stromal cell-derived HA on the BCC activities.

**Conclusion:**

These data suggested that HA in abnormal BM stroma might be a therapeutic candidate for bone metastasis of breast cancer.

**Video Abstract**

**Graphical abstract:**

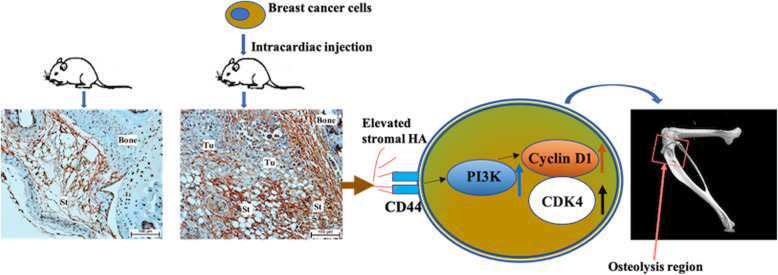

## Background

Malignant tumours are fatal diseases attributed to metastasis [1–3]. In breast cancer patients, bone is one of the most common sites for the localization of breast tumour cells [4, 5]. Breast cancer is incurable once bone metastases occur, and this condition often severely affect the patient’s quality of life [6]. Therefore, exploring the molecular mechanism of breast cancer distant metastasis and identifying new therapeutic target sites have become a research hotspot.

The bone marrow (BM) microenvironment contains many cytokines and high levels of extracellular matrix (ECM), which constitute the complex microenvironment of the BM. Hyaluronan (HA) is a nonprotein molecule in the BM matrix [7] and is one of the most abundant components of the ECM. The aberrant metabolism of HA plays an important role in tumorigenesis and development. For example, in the development of acute myeloid leukaemia, microenvironment remodelling resulting from the retention of HA in the BM matrix was significantly higher than that of normal BM matrix [8]. However, few studies have focused on how HA alters the tumour-associated BM microenvironment. In humans, HA can be produced by many cell types, and stromal cells are believed to be the predominant source of HA [9]. Whether HA from BM stroma is altered and its subsequent effects on the growth of metastatic breast cancer cells (BCCs) in bone need to be elucidated.

In this study, we utilized in vivo models to study the remodelling of HA in tumour-associated BM stroma. The abnormal metabolism of HA and its correlation with the malignant growth of BCCs and the modifications of intracellular signalling pathways were investigated by in vitro coculture of BM stromal cells with BCCs. After knockout of the HA receptor CD44 in BCCs, the receptor pathway of stromal HA on BCCs was investigated in vivo through intratibial inoculation and in vitro by coculture with HS5 cells.

## Materials and methods

### Animal experiment

Mice were obtained from Shanghai SLAC Laboratory Animal Co., Ltd. (China). All experimental protocols were approved by the appropriate institutional review board. Mice were subjected to tumour BM metastasis as previously described [10]. To model BM metastasis in mice, we delivered 7 × 10^5^ BCCs in 100 μL of PBS via intracardiac injection into the left ventricle of 4- to 6-week-old female nude mice. Nude mice aged 7–8 weeks were used for the intratibial experiments. For intratibial injection, the protocol was performed as previously described [11]. BCCs (3 × 10^5^) in 10 μL of PBS were injected into the tibias of the mice. The progression of bone destruction and the development of metastasis were monitored by micro-CT (Bruker, Germany).

### Cell lines

The metastatic bone-seeking human breast cancer cell line MDA-MB-231BO was a kind gift from Prof. Shen (Department of Oncology, Shanghai Jiao Tong University Affiliated Sixth People’s Hospital, Shanghai, China). The human BM stromal cell line HS5 and the highly malignant breast cancer cell line HS578T were obtained from the American Tissue Culture Collection (ATCC; USA). Cells were cultured at 37 °C in a humidified atmosphere in the presence of 5% CO_2_ and 95% air. MDA-MB-231BO and HS5 cell lines were cultured in high-glucose Dulbecco’s modified Eagle’s medium (DMEM; Gibco, USA) supplemented with 10% foetal bovine serum (FBS; Gibco), 100 units/mL penicillin (HyClone, USA), and 100 μg/mL streptomycin (HyClone). HS578T cells were maintained in high-glucose DMEM supplemented with 0.01 mg/mL human recombinant insulin, 10% FBS, 100 units/mL penicillin, and 100 μg/mL streptomycin. MDA-MB-231BO and HS578T cells were infected with GFP fluorescent protein. Lentiviral particles were purchased from Hanbio Company (China). The desired number of BCCs was plated in complete culture medium and infected with lentiviral supernatant. Stable clones expressing GFP were selected via puromycin dihydrochloride (Santa Cruz Biotechnology, USA).

### Immunofluorescence staining

HS5 cells were washed with PBS and fixed in cold methanol. After being fixed for 10 min at room temperature, the cells were blocked with 1% bovine serum albumin (BSA; Biofroxx, Germany) for 1 h and then stained with biotinylated a hyaluronan binding protein(HABP)antibody (Merck Millipore, Germany) at 4 °C overnight. Then, Alexa Fluor 488-conjugated streptavidin (Thermo Fisher Scientific, USA) and 4,6-diamidino-2-phenylindole (DAPI) were used to stain the cells. The hyaluronidase(HAase, Sigma-Aldrich, USA)-treated group was used as the control. The cells were imaged using a fluorescence microscope (Nikon, Japan).

### Particle exclusion assay

HS5 cells were cultured for 48 h in 48-well plates. Aldolized erythrocyte cells (BD, USA) were added to the medium and allowed to settle for 15 min at room temperature. The cells were viewed by phase-contrast microscopy. The pericellular HA matrix was evidenced by halos surrounding the cells from which the fixed red blood cells were excluded. Finally, the cells were treated with HAase for 5 min and imaged.

### Determination of cell proliferation in the coculture system

Coculture of cells was performed as previously described [12]. Briefly, HS5 BM stromal cells were seeded at 3 × 10^3^ cells/per well in 96-well plates (Cellvis, USA) and incubated overnight. GFP-positive BCCs were seeded alone or layered on HS5 cells. The fluorescence intensity (FI) per well was read at λex 488 nm/λem 528 nm to detect the numbers of GFP-tumour cells with a Synergy 4 multidetection microplate reader (BioTek, USA) at day 0 and day 3. The proliferation index was calculated by dividing FI in the same well at day 3/day 0 after subtracting the average of the background values of the corresponding condition (DMEM for GFP cultured alone or HS5 for cocultures).

### Western blot analysis

BCCs were sorted by flow cytometry. Western blot analysis was performed as previously described [13]. The efficiency of gene interference and the changes in cellular signalling molecules after CD44 gene interference were also tested by western blot analysis. Cells were lysed on ice with complete RIPA buffer mixture (Beyotime, China) composed of protease inhibitor and phosphatase inhibitor and then lysed for 45 min. The liquids were centrifuged at 15000 g for 10 min, and the supernatant was boiled with loading sample buffer (5×, Beyotime) for 5 min at 100 °C. Proteins from each group were separated by SDS-PAGE and transferred to PVDF membranes (Merck Millipore). Blocking was carried out using 5% milk in TBST buffer at room temperature for 1 h. The membranes were incubated at 4 °C overnight. The supplier and the specifications of each primary antibody were as follows: rabbit anti-PI3K polyclonal antibody, rabbit anti-CDK4 monoclonal antibody, rabbit anti-cyclin D1 monoclonal antibody, mouse anti-CD44 monoclonal antibody (Cell Signaling Technology, USA), and rabbit anti-HAS2 monoclonal antibody (Abcam, UK). We used GAPDH as an internal control for all analyses. The membranes were rinsed with TBST buffer three times. Subsequently, the membranes were incubated with HRP-conjugated anti-rabbit IgG (Liankebio, China) or HRP-conjugated anti-mouse IgG secondary antibodies (Liankebio) at room temperature for 1 h and washed three times with TBST. Immunoblotting was visualized using enhanced chemiluminescence solution (Merck Millipore), and a ChemiDocTM MP imaging system (Bio-Rad, USA) was used to detect protein expression. Bands were quantified using ImageJ software.

### HA and growth factor detection

The method was based on that of a previous report [14]. Before examination, the culture medium was centrifuged at 8000 g for 10 min. The HA levels were determined by CLIA (New Industries Biomedical Engineering, China). The total protein content was measured using a BCA Protein Assay Kit (Thermo Fisher Scientific). The HA concentrations in the cell culture medium were routinely normalized to the total protein content of the cell after 72 h of culture and are expressed as nanogram (ng)/microgram (μg) total protein per 72 h. HA extraction was performed as previously described [15]. The BM of the mouse tibia was immediately collected after sacrificing the animals. After digestion with proteinase K, DNase and RNase (Sigma-Aldrich), the HA pellet was dissolved in 500 μL of distilled water and incubated at 4 °C. Aliquots were treated with 20 mg/mL HAase, incubated overnight at 37 °C, and boiled at 100 °C. Growth factors, including TGF β1, PDGF-BB, EGF, and IGF, were assayed by ELISAs according to the manufacturer’s instructions (R&D, USA).

### HA analysis by gel electrophoresis

Twenty-five microlitres of each sample was mixed with 5 μL of DNA loading solution (Thermo Fisher Scientific) and loaded onto a 1% agarose gel (Amresco, USA). Ten microlitres of HA molecular size markers, which included markers at 15 MD, 200 kD and 10 kD, respectively, was run to determine the size of HA from each sample. The gel was placed in a 0.005% (w/v) Stains-All (Sigma-Aldrich) in 50% ethanol solution overnight. For destaining, the gel was placed in distilled water for 24 h in the dark, placed under natural light in distilled water for 1 h to complete the final destaining stages and then photographed under white light.

### Cell growth assay

Cell growth was determined using the Cell Counting Kit-8 (CCK-8, Dojindo, China) assay. Briefly, cells were seeded into each well of a 96-well plate and incubated at 5% CO_2_ and 37 °C for 72 h. Ten microlitres of CCK-8 reagent was added to each well at the endpoint, and the plates were incubated for an additional 2 h at 37 °C. Finally, the absorbance of the control and experimental groups at 450 nm was measured with a spectrophotometer (Bio-Rad).

### Quantitative real-time PCR (qRT-PCR)

Total RNA from cultured cells was extracted using TRIzol according to the manufacturer’s instructions. The RNA quality and quantity were measured using a NanoDrop 2000 spectrophotometer (Thermo Fisher Scientific). For analysis of the expression of hyaluronan synthases (HAS), RNA samples were reverse transcribed using the PrimeScript™ RT reagent Kit (TaKaRa, China), and the relative mRNA expression levels were determined by qRT-PCR using TB Green Premix Ex Taq II (TaKaRa). GAPDH was used as an internal control. Primer sequences were as follows: HAS1, GGAATAACCTCTTGCAGCAGTTTC/GCCGGTCATCCCCAAAAG; HAS2, TCGCAACACGTAACGCAAT/ACTTCTCTTTTTCCACCCCATTT; HAS3, AACAAGTACGACTCATGGATTTCC/AGGCCAATGAAGTTCACCAC; GAPDH, CCCATGTTCGTATGGGTGT/TGGTCATGATCCTTCCACGATA. All qRT-PCR reactions were performed in a 7500 Real-Time PCR system (Applied Biosystems, Thermo Fisher Scientific).

### RNAi

Double-stranded siRNA targeting HAS2 and control siRNA (catalogue number: siN05815122147) were designed and synthesized by RiboBio Co. (China). The corresponding target mRNA sequences for the siRNA were as follows:

si-HAS2, GTATCAGTTTGGTTT. HS5 cells (3.5 × 10^5^) were plated in 6-well plates. Twenty-four hours later, the HS5 cells were transfected with siRNA according to the manufacturer’s protocol when cells reached 50% confluence. Briefly, 5 μL of HAS2 siRNA (20 μmol/L) was diluted in 150 μL of Opti-MEM reduced serum medium (Opti-MEM, Thermo Fisher Scientific) and mixed with 9 μL of Lipofectamine RNAiMAX transfection reagent (Thermo Fisher Scientific) prediluted in 150 μL of Opti-MEM. After a 20 min incubation at room temperature, the complexes were added to the cells. Cells were transfected for 48 h. HS5 cells transfected with a nontarget control siRNA were used as controls. The RNAi results were evaluated by qPCR and western blots.

### CD44 gene interference

Oligonucleotides encoding short hairpin RNAs (shRNA) that target standard exons of CD44 were cloned into the plasmid vector Plkd-cmv-GFP-hU6 Puro (OBio, China). The plasmids were transfected into BCCs, and the cells were cultured for 2 weeks in the presence of puromycin (4 μg/mL; Sigma-Aldrich). BCCs transfected with Plkd-cmv-GFP-hU6-control shRNA were used as controls. The shRNA results were evaluated by western blots.

The CD44 CRISPR/Cas9 KO plasmid, HDR plasmid and control CRISPR/Cas9 plasmid were purchased from Santa Cruz Biotechnology. BCCs (5 × 10^4^) were plated on 24-well plates. After 24 h of incubation, the cells were transfected with CRISPR/Cas9 plasmids according to the manufacturer’s protocol when they reached 60% confluence. The ratio of the target plasmid to the transfection reagent was 0.5 μg/2.5 μL. After 25 min of incubation at room temperature, the complexes were added to the cells. After the cells were expanded, cells with positive expression were sorted, and the effects of CD44 knockout were evaluated by western blots.

### Immunohistochemistry (IHC)

For histological analyses of induced bone destruction lesions, IHC was performed on decalcified and paraffin sections. Tibial bones of nude mice were excised and fixed in 10% neutral-buffered formalin. The bone was decalcified, embedded in paraffin, sectioned, and stained. The expression of HA was indicated by detecting HABP in bone sections. Histological sections underwent 3-amino-9-ethyl-carbazole (AEC, Dako, Denmark) IHC staining for HABP. HA expression was evaluated using ImageJ software.

### Statistical analysis

Statistical analysis and image production were performed using GraphPad Prism 6.0 and Adobe Illustrator CC 2018. Data are expressed as the mean ± SEM. Unpaired Student’s *t* test was used to compare two samples, and *p* < 0.05 was considered statistically significant. Survival analysis was performed between two groups to compare the differences. The occurrence of bone destruction was set to “1”, and boneless destruction was set to “0”. At different time points of scanning, the number of animals with bone destruction was indicated by the number “1”, and the number of animals without bone destruction was indicated by the number “0”. Group comparisons were performed using the Mantel-Cox test. When *p* < 0.05, there was a significant difference between the two groups.

## Results

### Abnormal metabolism of HA in the BM stromal microenvironment

**Fig. 1:**
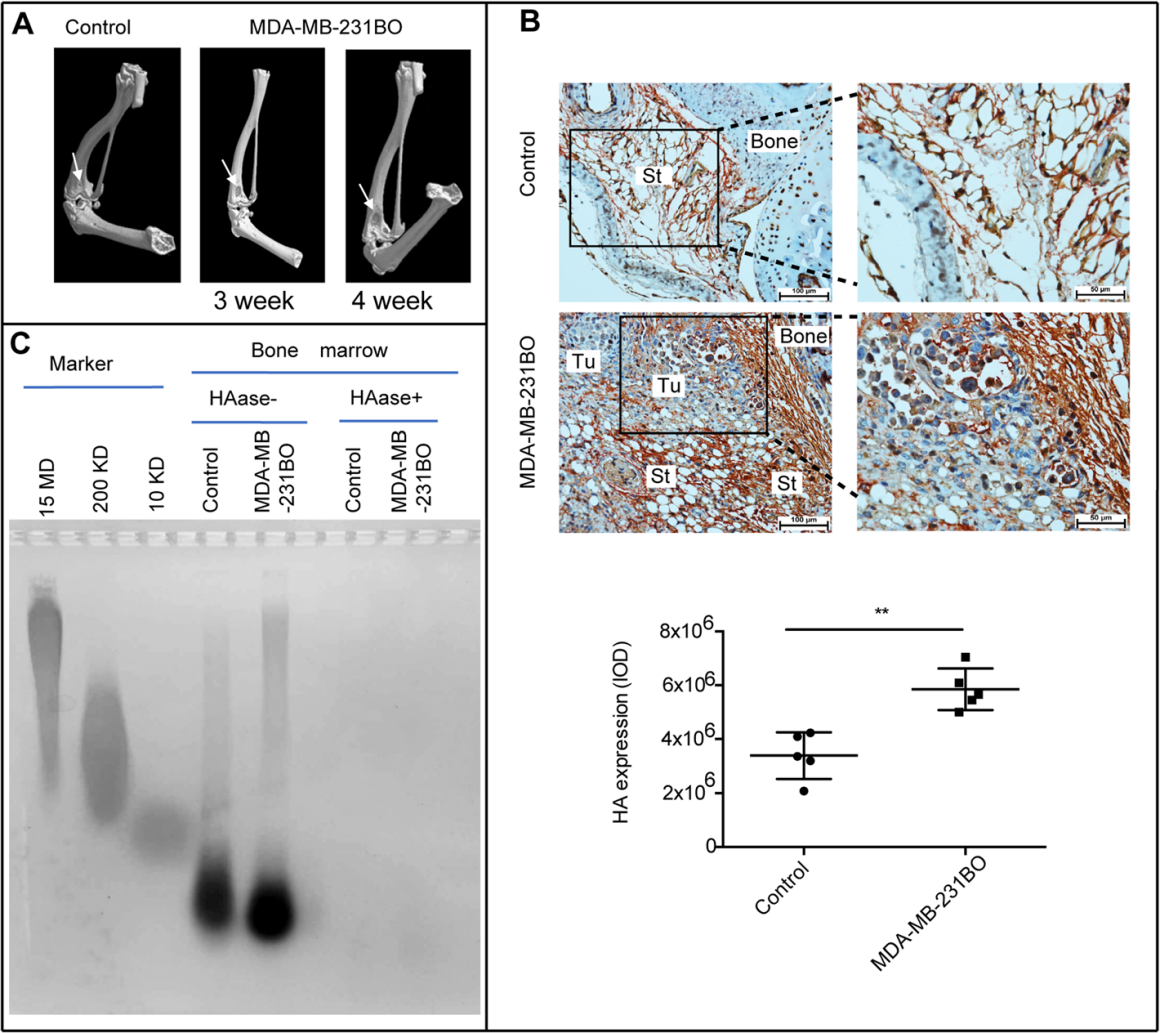
Elevated deposition of HA in BM stroma. **a.** Micro-CT was used to scan the bone after MDA-MB-231BO cell intracardiac injection into the left ventricle of 4- to 6-week-old female nude mice. Arrows indicate the osteolytic areas in the control and treated groups. **b.** Immunohistochemical expression of HA in BM. Brown indicates HA. Representative images of each group are shown. Left scale bars, 100 μm; right scale bars, 50 μm. Tu: tumour, St: stroma. Scatter plots showing the levels of HA. **c.** Elevated HA in breast cancer-associated BM stroma was analysed by electrophoresis

To explore whether HA in tumour-metastasized BM was abnormally metabolized, we constructed a model of breast cancer bone metastasis by injecting MDA-MB-231BO cells into the left ventricle of the mice. Osteolytic areas were confirmed by micro-CT scanning of the tibias of mice. There was obvious bone destruction in the BCC-injected group compared with the control group at 3 and 4 weeks (Fig. 1a). Normally, HA indicated by HABP staining was mainly distributed in the BM stroma (Fig. 1b). In experimental mice, the accumulation of HA in the BM stroma adjacent to bone was obviously increased after injection of MDA-MB-231BO cells into the left ventricle for 4 weeks (Fig. 1b). HA from the BM was analysed by gel electrophoresis. Notably, the HA level was abnormally increased in the MDA-MB-231BO-treated group compared with the control group (Fig. 1c).

### Bone marrow HS5 stromal cells promoted BCC growth by enhancing proliferative signalling pathways

To verify the effects of the abnormal deposition of HA in the tumour-associated BM stroma on BCCs, we cocultured HS5 cells with high expression of HA (Fig. S1A and B) with BCCs in vitro.

**Fig. 2:**
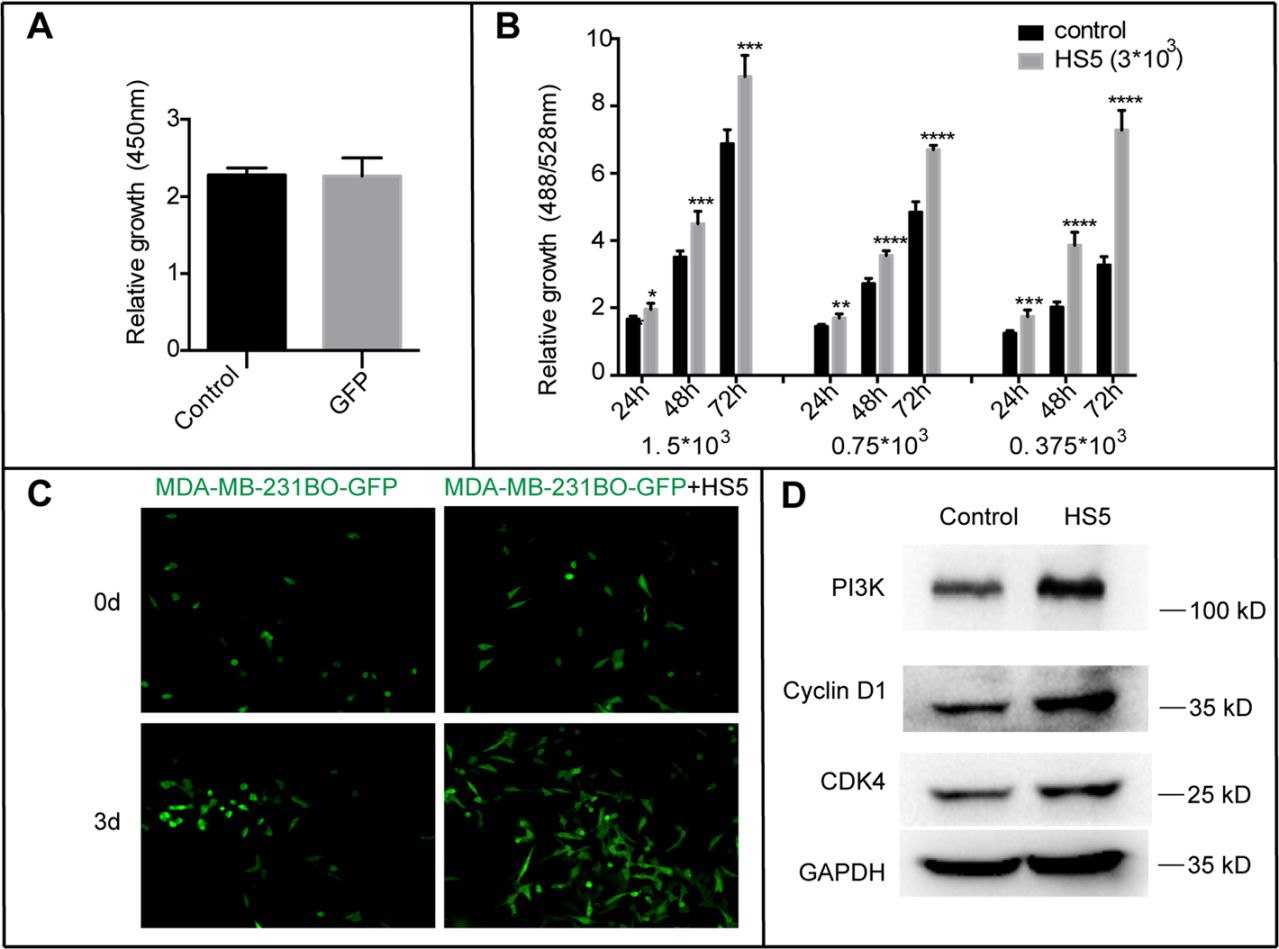
The BM stromal cell line HS5 promoted MDA-MB-231BO-GFP cell proliferation. **a.** The effect of GFP protein on the growth of MDA-MB-231BO cells was analysed by CCK-8 assays. **b.** The growth of MDA-MB-231BO-GFP cells after coculturing with HS5 stromal cells was evaluated by detecting the fluorescence intensity of GFP. The asterisk indicates that the relative growth of MDA-MB-231BO-GFP cells in the coculture environment was significantly promoted compared with that of the control group of MDA-MB-231BO-GFP cells alone. Bars represent the mean ± SEM (*p < 0.05, **p < 0.01, ***p < 0.005 and ****p < 0.0001 by unpaired Student’s *t* test). **c.** Fluorescence graphs show the number of MDA-MB-231BO-GFP cells after coculture with stromal HS5 cells or culture alone for 0 and 3 days. **d.** After coculture with HS5 cells for three days, the proliferation-related proteins PI3K, Cyclin D1, and CDK4 in MDA-MB-231BO-GFP cells were detected by western blots

BCCs infected with GFP fluorescent protein did not affect the growth of the cells themselves, as shown in Fig. 2a. In Fig. 2b, the results showed that HS5 cells could promote the growth of BCCs at either a low ratio (MDA-MB-231BO:HS5) of 1:2, a ratio of 1:4 or a high ratio of 1:8 at 24 h, 48 h, and 72 h, respectively. This promotion was particularly evident in the ratio of BCCs to stromal cells at 1:8 for 3 days of coculture. Figure 2c indicates that the number of MDA-MB-231BO-GFP cells was increased in the microenvironment matrix composed of HS5. To further investigate the downstream signalling pathways related to the promotion, we performed western blotting to detect PI3K, Cyclin D1, and CDK4 expression. After coculture with HS5 cells for 3 days, the expression levels of the PI3K, Cyclin D1, and CDK4 proteins were significantly increased in the MDA-MB-231BO-GFP cells under HS5 coculture conditions (Fig. 2d). Taken together, these results showed that the HS5 cell matrix could promote the growth of MDA-MB-231BO breast cancer cells by enhancing proliferative signal transduction. To further confirm these results, we performed the same experiment on an additional malignant breast cancer cell line, HS578T. First, HS578T cells were infected with GFP fluorescent protein and showed no changes in cell growth (Fig. S2A). After coculture with HS5 cells, the growth of the HS578T-GFP cells was promoted at either a low ratio (HS578T:HS5) of 1:2, a ratio of 1:4 or a high ratio of 1:8 at 24 h, 48 h, and 72 h, respectively (data are shown in Fig. S2B).

### HA mediated the growth of BCCs in the BM matrix microenvironment

**Fig. 3:**
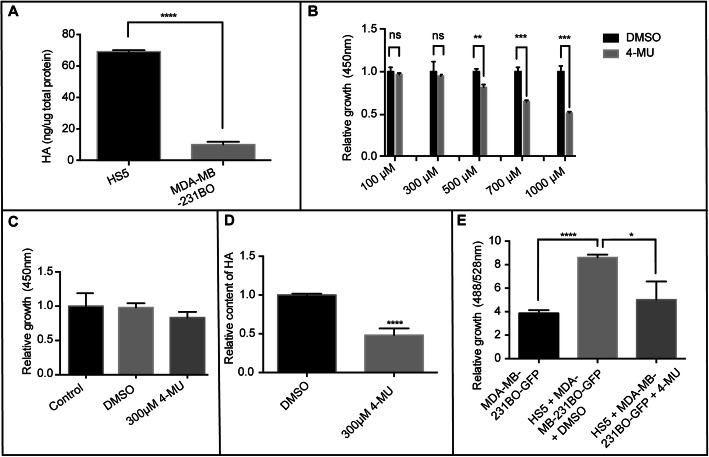
HA mediated the proliferation of MDA-MB-231BO-GFP cells in the matrix microenvironment. **a.** The HA content in the culture supernatant was assayed by CLIA. (****p < 0.0001). **b.** The growth of HS5 cells was detected by CCK-8 assays after adding 100 μmol/L, 300 μmol/L, 500 μmol/L, 700 μmol/L, and 1000 μmol/L 4-MU for 3 days. (**p < 0.01 and ***p < 0.005 compared with the control group as measured by unpaired Student’s *t* test). **c.** The growth of MDA-MB-231BO cells was detected by CCK-8 assays after adding 300 μmol/L 4-MU for 3 days. **d.** HS5 cells were pretreated with 300 μmol/L 4-MU. Twenty-four hours later, MDA-MB-231BO-GFP cells were added. Furthermore, treatment with 300 μmol/L 4-MU was continued in the coculturing system for 3 days. The HA content in the supernatants was determined by CLIA. (****p < 0.0001 compared with the control group as measured by unpaired Student’s *t* test). **e.** The fluorescence intensity of GFP was used to measure the growth of MDA-MB-231BO-GFP cells, which were separately treated with DMSO alone or cocultured with stromal HS5 cells before and after treatment with 300 μmol/L 4-MU. (*p < 0.05 and ****p < 0.0001 compared with the control group as measured by unpaired Student’s *t* test). Bars represent the mean ± SEM

Next, we examined the remodelling of HA and its effects on the growth of BCCs. The culture supernatants of HS5 cells and MDA-MB-231BO cells were collected at 72 h. The HA content was assayed as described previously (Fig. 3a). The results showed that compared to the BCCs, the HS5 stromal cells secreted more HA, suggesting that the HA in coculture was mainly derived from the HS5 stromal cells.

To explore whether the abnormal accumulation of HA could affect the growth of BCCs, we inhibited the synthesis of HA in coculture with an inhibitor of HA synthesis, 4-methylumpholone (4-MU), which inhibits HA synthesis mainly through two aspects: inhibition of the production of HAS, especially HAS2 and HAS3, at the post-transcriptional level [16, 17] and inhibition of the synthesis of UDP-GlcUA, which is a prerequisite for HA [16]. Different concentrations of 4-MU were applied to HS5 cells to determine the toxic effect, and the maximum working concentration of 4-MU, 300 μmol/L of 4-MU (Fig. 3b), was selected, which had no toxic effects (Fig. 3c). After treatment with 4-MU, the HA content was significantly reduced (Fig. 3d), and the proliferation of BCCs was significantly attenuated (Fig. 3e). Our data indicated that the inhibition of BCC growth was mediated by HA within the culture context.

### Altered expression of HA in stromal cells affected the growth of BCCs

**Fig. 4:**
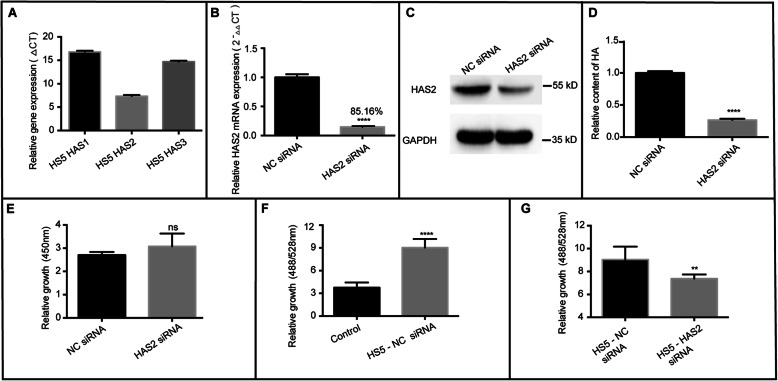
HA derived from HS5 stromal cells affected the growth of MDA-MB-231BO-GFP cells. **a.** The expression of three HA synthases, HAS1, HAS2, and HAS3, in HS5 cells was detected by qPCR. **b.** The interference efficiency of HAS2 siRNA was evaluated by qPCR in HS5 cells. **c.** The expression of HAS2 was detected by western blots after interference with HAS2 siRNA in HS5 cells. **d.** HA content in the supernatant was determined after HAS2 expression was downregulated in HS5 cells. (****p < 0.0001 compared with the NC siRNA group as measured by unpaired Student’s *t* test). **e.** The growth of HS5 cells after downregulation of HAS2 was detected by CCK-8 assays. **F.** A total of 3000 HS5-NC siRNA cells/well were plated in special 96-well plates. Twenty-four hours later, 0.375 × 10^3^ MDA-MB-231BO-GFP cells were plated in the HS5 cell wells. MDA-MB-231BO-GFP cells were cultured alone as controls. After three days of culture, the fluorescence intensity of GFP was determined (****p < 0.0001 compared with the control group as measured by unpaired Student’s *t* test). **G.** After HAS2 in HS5 cells was knocked down by HAS2 siRNA, the cells were cocultured with MDA-MB-231BO-GFP cells for 72 h. The fluorescence intensity of GFP was measured. (**p < 0.01 compared with the HS5-NC siRNA group as measured by unpaired Student’s *t* test). Bars represent the mean ± SEM

HAS is the main functional enzyme of HA synthesis in cells and includes HAS1, HAS2, and HAS3 [18, 19]. We further explored whether BM stromal cell-derived HA promotes the proliferation of BCCs by decreasing the expression of HA in HS5 cells. We first found that the mRNA expression of HAS2 was significantly higher than that of HAS1 and HAS3, as shown in Fig. 4a. Next, we transfected siRNA targeting HAS2 and control siRNA into HS5 stromal cells. The data showed that HAS2 expression was significantly reduced by 85.16% at the mRNA level (Fig. 4b), following a significant parallel decrease in HAS2 protein expression (Fig. 4c). Figure 4d shows that HAS2 siRNA significantly decreased the amount of HA secreted by the cells but did not affect the growth of HS5 cells compared to control siRNA (Fig. 4e). Then, the HS5 cells were cocultured with BCCs after HAS2 siRNA interference. Figure 4f and Fig. S3A show that the control siRNA did not influence the effects of HS5 on the proliferation of MDA-MB-231BO-GFP and HS578T-GFP cells. However, the growth of BCCs was significantly attenuated after coculture with HAS2 knockdown HS5 cells (Fig. 4g and Fig. S3B), suggesting that the expression of HA in stromal cells plays a role in mediating BCC proliferation. We also found that the proliferation-promoting effect of HS5 could not be completely eliminated after HAS2 siRNA interference. It is possible that HA synthesis could not be completely inhibited since HS5 contains synthases other than HAS2. Moreover, HS5 supernatant contains some active growth factors, such as TGF β1, PDGF-BB, EGF, and IGF (Fig. S4), which may also contribute to growth.

### The effects of the HA receptor CD44 on the growth of MDA-MB-231BO cells in vitro and the progression of osteolytic lesions in bone

**Fig. 5:**
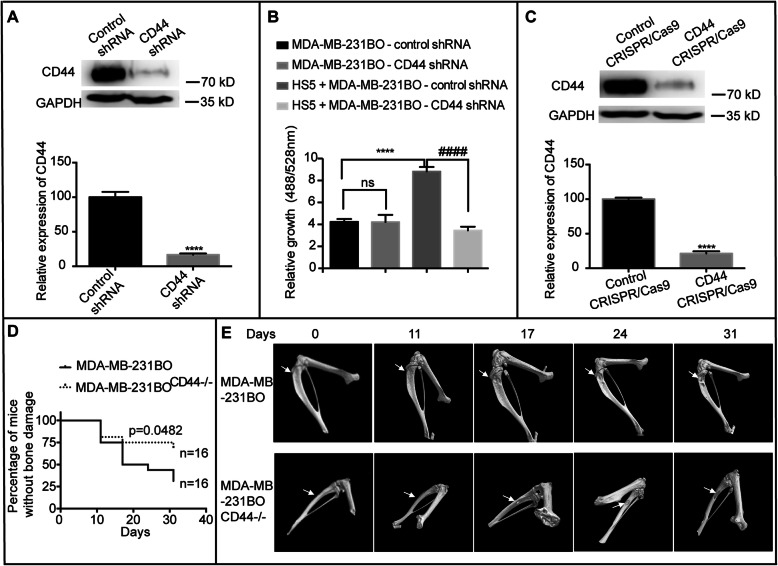
HA promoted the growth of MDA-MB-231BO cells via the CD44 receptor. **a.** The knockdown effect of CD44 shRNA on MDA-MB-231BO cells was detected by western blots. The band intensities were analysed by densitometry analysis (****p < 0.0001 compared with the control shRNA group as measured by unpaired Student’s *t* test). **b.** MDA-MB-231BO-CD44 shRNA cells and MDA-MB-231BO-control shRNA cells were cocultured with HS5 cells for 72 h. The fluorescence intensity of GFP was measured. Data are presented as the mean ± SEM (****p < 0.0001 compared with the MDA-MB-231BO-control shRNA cells alone group as measured by unpaired Student’s *t* test, #### *p* < 0.0001 compared with the group of HS5 cells cocultured with MDA-MB-231BO-control shRNA cells as measured by unpaired Student’s *t* test). **c.** The knockout efficiency of CD44 in MDA-MB-231BO cells was detected by western blots. The band intensities were analysed by densitometry analysis (****p < 0.0001 compared with the control CRISPR/Cas9 group as measured by unpaired Student’s *t* test). **d.** MDA-MB-231BO^CD44−/−^ cells and MDA-MB-231BO cells were injected into the tibia of mice, and the tibias were monitored on days 0, 11, 17, 24 and 31. *n* = 16 (*p* = 0.0482 compared with the MDA-MB-231BO-treated group by the Mantel-Cox test). **e.** The micro-CT images showed the bone destruction of the two groups at days 0, 11, 17, 24, and 31 after the injection. Arrows indicate the site of bone damage

CD44 is the major receptor of HA, and most of the activities of HA on cells are mediated through CD44 [20]. To investigate whether CD44 promoted BCC proliferation in a bone microenvironment, we knocked down the expression of CD44 in MDA-MB-231BO cells through shRNA lentivirus (Fig. 5a). Knockdown of CD44 did not affect the growth of MDA-MB-231BO cells (Fig. 5b and Fig. S5A) or related signalling pathways (Fig. S5B). As a result, in coculture experiments, HS5 stromal cells showed no effects on the proliferation of MDA-MB-231BO-CD44 shRNA cells (*p* < 0.0001) (Fig. 5b). To further verify that the effect of HA on BCCs was through the CD44 signalling pathway, we knocked out CD44 by CRISPR (Fig. 5c) and injected the CD44^−/−^ cells into mice through the tibia. Figure 5d shows that the number of animals that underwent bone destruction in the MDA-MB-231BO^CD44−/−^ cell injection group was less than that in the control group. Figure 5e shows the micro-CT images of tibial destruction and demonstrates that the tibial destruction of the MDA-MB-231BO^CD44−/−^ cell injection group was weaker than that of the MDA-MB-231BO cell injection group. Supplementary Table lists the specific number of bone destructions in the two groups. The results further demonstrated that the remodelling of HA in the stroma may modulate metastatic BCC growth through the receptor CD44.

## Discussion

The growth of tumour cells from primary lesions to micrometastases in distant organs is the leading cause of death in cancer patients [2], in which the microenvironment of metastatic organs plays a vital role [21]. For breast cancer, bone is the main metastatic site, and the BM microenvironment provides a supportive environment to regulate the progression of tumour cells [22]. HA is one of the most abundant ECM components enriched in the BM microenvironment [23]. Under normal conditions, the metabolism of ECM is physiologically constant, and the synthesis and degradation of HA are balanced. However, during the progression of pathological destruction, such as cancer, HA may be abnormally metabolized, remodelling the microenvironment of the ECM. For instance, in the development of acute myeloid leukaemia, the retention of HA in the BM matrix was significantly higher than that of normal BM matrix [8]. At present, it is still unknown how stromal HA is metabolized in tumour metastatic BM and to what extent HA remodelling modulates cancer cell malignancy. In this study, we first established a bone metastatic model through intracardiac injection with highly bone metastatic BCCs, MDA-MB-231BO cells. The results showed that stromal HA in the BM was significantly increased after injection, which was identical to the findings of other studies [24, 25]. These reports showed that in mouse breast cancer xenograft models, HA accumulation in bone metastatic lesions was perturbed peripherally, and administration of either 4-MU or oligosaccharides of hyaluronan (oHA) decreased the expression of stromal HA and inhibited the expansion of osteolytic lesions [24, 25].

As there are no reports on the direct effects of bone stromal HA on breast cancer growth, we next developed an in vitro model to mimic the BM microenvironment for an HA remodelling study, in which a BM-derived stromal cell line, HS5, was selected for its high expression of HA. We found that HS5 cells could promote the growth of BCCs in coculture through the activation of PI3K signalling transduction, which is an important pathway in the proliferation of tumour cells [26]. Moreover, the cell cycle regulators cyclin D1 and CDK4, which promote cell cycle progression [27–29], were obviously elevated in the BCCs. These results indicated that HS5 provided an environment conducive to cancer cell proliferation.

Previous studies have indicated that in breast cancer bone metastasis, HA from cancer stem cells promoted the expression of PDGF-BB in tumour-associated macrophages, induced the activation of HS5 cells and promoted the growth of breast cancer stem-like cells [30]. In our experiments, the HA levels secreted by HS5 were significantly higher than those secreted by BCCs; therefore, we focused on whether HS5 stromal cell-derived HA is associated with breast cancer cell growth. When HA expression was inhibited by 4-MU, a widely recognized specific inhibitor of HA synthesis, in the coculture environment, the promotion of HS5 cells on BCCs was significantly decreased, implying that HA played an important role in promoting the proliferation of BCCs. Importantly, when HAS2 was inhibited in HS5 cells, the secretion of HA was substantially reduced, and the proliferative ability of the BCCs was thus weakened. The results indicated that HA derived from HS5 cells played a key role in promoting the growth of BCCs. However, the reduction of HA in the coculture environment did not completely abolish the promotion of the proliferation of MDA-MB-231BO cells, suggesting that HA synthesis might not be completely inhibited after silencing of the HAS2 gene or HA may not be the only factor involved in the proliferative effect on BCCs. As we demonstrated in the supplements, some proliferation-promoting factors, such as TGF β1, PDGF-BB, EGF, and IGF, are detectable in the culture supernatants of HS5 cells. These cytokines must be active in our coculture system. However, our present study focused on HS5-derived HA on BCC proliferation, which was confirmed by both 4-MU inhibition and HAS2 interference experiments. The results suggest the effects of stromal HA on cell growth but cannot exclude the effects of other cytokine activities. Further detailed studies are needed.

Moreover, the difference in molecular weight and tissue content can lead to differences in HA activity [31, 32]. It is now accepted that high molecular weight HA (HMW-HA) usually inhibits cell proliferation [33], while low molecular weight HA (LMW-HA) may promote proinflammatory events [34, 35]. Our previous study also showed that simply concentrated HMW-HA can arrest breast cancer cell cycle progression, which seems to be contradicted by the present study [36]. Such diversity may be due to HA MW heteropolysaccharides and tissue distribution; for example, there are thousands of HA molecules of different sizes in tumour remodelling microenvironments. The present study on HS5-derived HA includes various fragments of HA that mostly mimic the in vivo microenvironments, therefore resulting in a promotion of cancer cells. Others have also shown that accumulation and increased levels of HA contribute to tumour progression [20]. Overall, it is worth further investigating the details of HA in cancer progression and metastasis, especially the abnormal metabolism of HA in tumour microenvironments.

Most of the functions of HA are mediated through binding to the cell receptor, and CD44 is the main receptor for HA [20]. The binding of HA to CD44 induces conformational changes to the receptor as well as post-translational modifications that regulate downstream signalling pathways, resulting in numerous pathobiological processes, including inflammation, wound healing, tumour growth, and metastasis [37–40]. In this study, CD44 was first knocked down in BCCs, and coculture experiments with HS5 cells were repeated as before. The results indicated that the HS5 cells showed no effects on BCC growth after the downregulation of CD44 expression. Because CD44 can influence the metastasis and invasion of cancer cells [41, 42], we next performed an in vivo experiment with CD44 knockout BCCs directly injected into the mouse through the tibia instead of the left ventricle. The results showed that the number of mice showing osteolytic bone metastasis and the extent of bone destruction in the CD44^−/−^ cell injection group were significantly decreased. The data suggested that the effects of HA-mediated bone microenvironment remodelling after BCC infiltration may be conducted through binding to CD44.

## Conclusions

In summary, our results showed that stromal HA in tumour-associated BM was abnormally distributed and that remodelling of the microenvironment may influence the growth of BCCs. The effects of HA on cancer cells may be mediated through its receptor CD44, which triggers a cascade of signal transduction increases, including increases in PI3K, Cyclin D1 and CDK4. Notably, the abnormal deposition of HA was mainly derived from tumour-associated BM stroma. These findings suggest the potential of stromal HA in bone as a target for treating bone metastasis of breast cancer.

## Supplementary information

**Additional file 1: Figure S1.** The high expression of HA in HS5 cells. **A.** The expression of HA on HS5 cells was analysed by immunofluorescence. Green indicates HA and blue represents DAPI. **B.** HS5 cells were cultured for 48 h. HA in pericellular matrix was visualized by particle exclusion. The results showed that HS5 cells highly expressed HA on the surface and formed pericellular HA matrix.

**Additional file 2: Figure S2.** BM stromal cell HS5 promoted HS578T-GFP cells proliferation. A. The effect of GFP protein on the growth of HS578T cells was determined by CCK-8 assays. B. The growth of HS578T-GFP cells after coculturing with stromal cell HS5 was evaluated by detecting the fluorescence intensity of GFP. The asterisk indicates that the relative growth of HS578T-GFP cells in the coculture environment was significantly promoted compared with that of the control group of HS578T-GFP alone. Bars represent mean ± SEM. (*p < 0.05, **p < 0.01, ***p < 0.005 and ****p < 0.0001 by unpaired Student’s *t* test).

**Additional file 3: Figure S3.** HA derived from stromal cell HS5 affected the growth of HS578T-GFP cells. **A.** A total of 3000 HS5-NC siRNA cells/well were plated in special 96-well plates. Twenty-four hours later, 0.375 × 10^3^ HS578T-GFP cells were plated in the HS5 cell wells. HS578T-GFP cells were cultured alone as controls. After three days of culture, the fluorescence intensity of GFP was determined, (****p < 0.0001 compared with control group as measured by unpaired Student’s *t* test). **B.** After HAS2 in HS5 cells was knocked down by HAS2 siRNA, the cells were cocultured with HS578T-GFP cells for 72 h. The fluorescence intensity of GFP ​​was measured. (***p < 0.005 compared with HS5-NC siRNA group as measured by unpaired Student’s *t* test). Bars represent mean ± SEM.

**Additional file 4: Figure S4.** Levels of cytokines measured from HS5 culture supernatants. Various cytokines in the culture supernatants were measured by ELISAs.

**Additional file 5: Figure S5.** The effect of CD44 on breast cancer cell growth. **A.** CCK-8 proliferation assay was used to detect the growth of MDA-MB-231BO cells after downregulation of CD44. Bars represent mean ± SEM. **B.** Western blot was used to detect the expression of signalling proteins, including PI3K, Cyclin D1, and CDK4 after downregulation of CD44.

**Additional file 6: Table 1.** The number of mice of osteolysis.

## Data Availability

The datasets used and/or analysed during the current study are available from the corresponding author on reasonable request.

## Abbreviations

BM: Bone marrow
HA: Hyaluronan
BCCs: Breast cancer cells
ECM: Extracellular matrix
ATCC: American Tissue Culture Collection
DMEM: Dulbecco’s modified Eagle’s medium
FBS: Foetal bovine serum
FI: Fluorescence intensity
HAase: Hyaluronidase
CCK-8: Cell Counting Kit-8
HAS: Hyaluronan synthase
shRNA: Short hairpin RNA
IHC: Immunohistochemistry
AEC: 3-amino-9-ethyl-carbazole
MW: Molecular weight
oHA: Oligosaccharides of hyaluronan
4-MU: 4-methylumpholone
