# Supplementary figures and images for: Remodelling of the bone marrow microenvironment by stromal hyaluronan modulates the malignancy of breast cancer cells
Source: Cell Commun Signal. 2020 Jun 9;18:89. doi: 10.1186/s12964-020-00592-z (PMC7285718; doi:10.1186/s12964-020-00592-z)

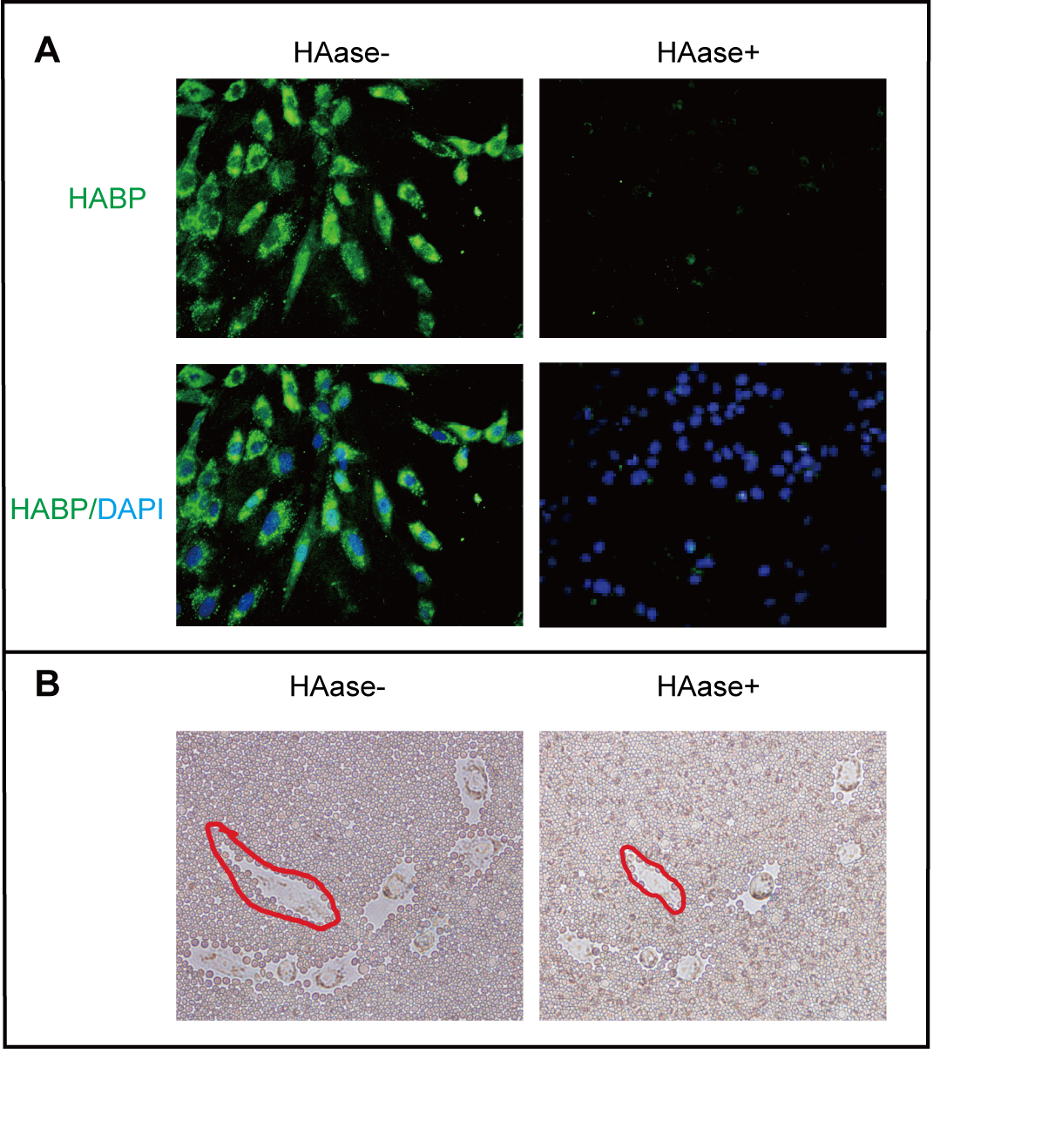

Supplement: Supplementary file 2 — Additional file 1: Figure S1. The high expression of HA in HS5 cells. A. The expression of HA on HS5 cells was analysed by immunofluorescence. Green indicates HA and blue represents DAPI. B. HS5 cells were cultured for 48 h. HA in pericellular matrix was visualized by particle exclusion. The results showed that HS5 cells highly expressed HA on the surface and formed pericellular HA matrix. [file 12964_2020_592_MOESM2_ESM.tif]

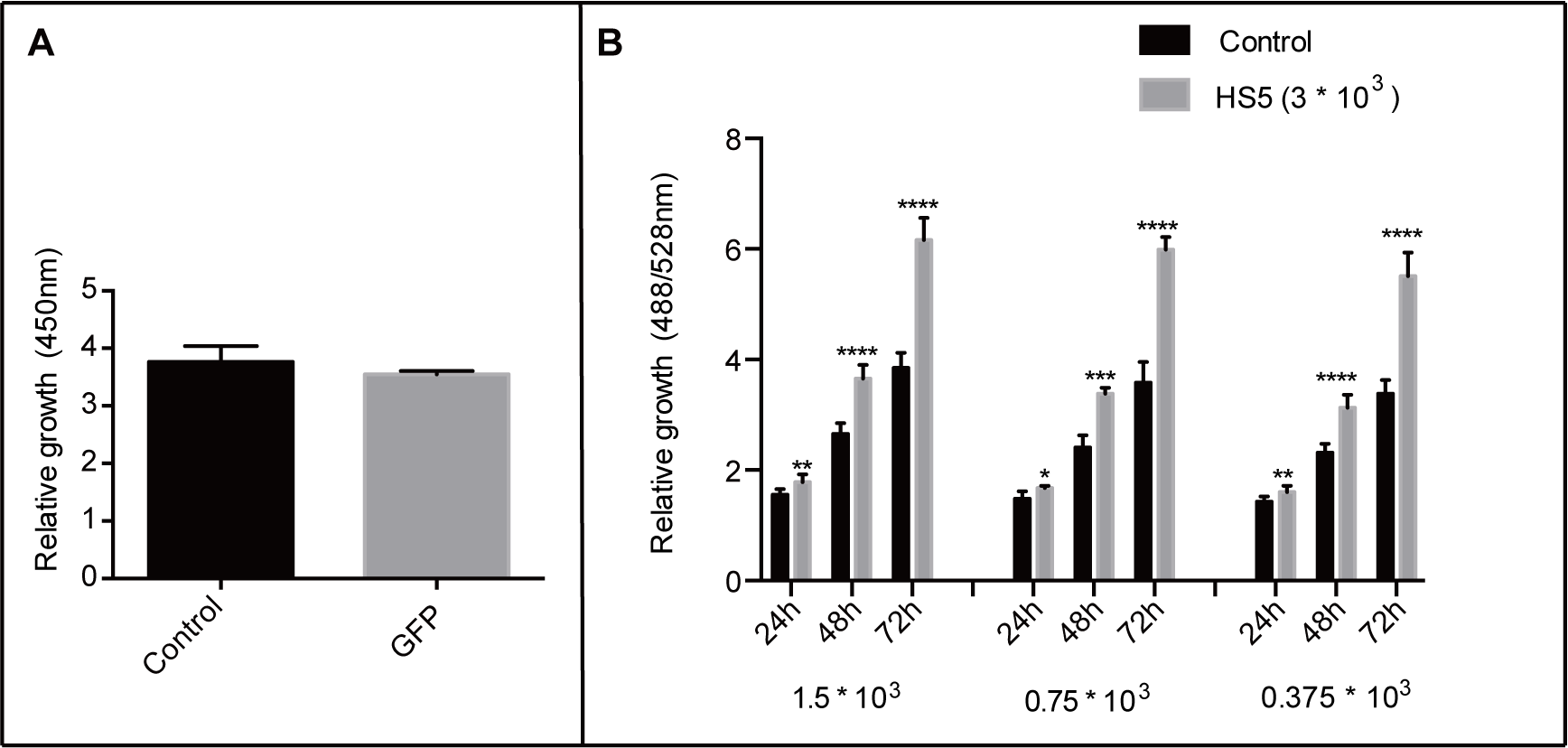

Supplement: Supplementary file 3 — Additional file 2: Figure S2. BM stromal cell HS5 promoted HS578T-GFP cells proliferation. A. The effect of GFP protein on the growth of HS578T cells was determined by CCK-8 assays. B. The growth of HS578T-GFP cells after coculturing with stromal cell HS5 was evaluated by detecting the fluorescence intensity of GFP. The asterisk indicates that the relative growth of HS578T-GFP cells in the coculture environment was significantly promoted compared with that of the control group of HS578T-GFP alone. Bars represent mean ± SEM. (*p < 0.05, **p < 0.01, ***p < 0.005 and ****p < 0.0001 by unpaired Student’s t test). [file 12964_2020_592_MOESM3_ESM.tif]

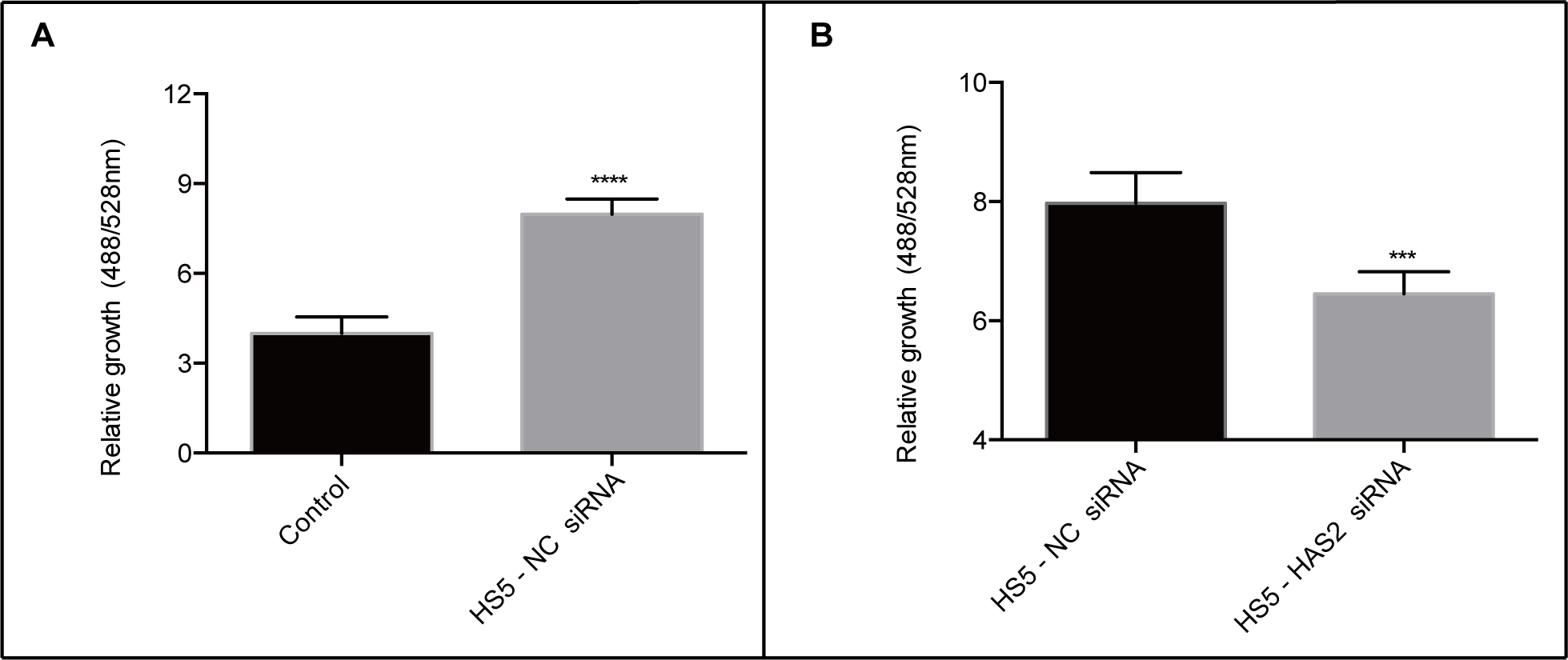

Supplement: Supplementary file 4 — Additional file 3: Figure S3. HA derived from stromal cell HS5 affected the growth of HS578T-GFP cells. A. A total of 3000 HS5-NC siRNA cells/well were plated in special 96-well plates. Twenty-four hours later, 0.375 × 10^3^ HS578T-GFP cells were plated in the HS5 cell wells. HS578T-GFP cells were cultured alone as controls. After three days of culture, the fluorescence intensity of GFP was determined, (****p < 0.0001 compared with control group as measured by unpaired Student’s t test). B. After HAS2 in HS5 cells was knocked down by HAS2 siRNA, the cells were cocultured with HS578T-GFP cells for 72 h. The fluorescence intensity of GFP ​​was measured. (***p < 0.005 compared with HS5-NC siRNA group as measured by unpaired Student’s t test). Bars represent mean ± SEM. [file 12964_2020_592_MOESM4_ESM.tif]

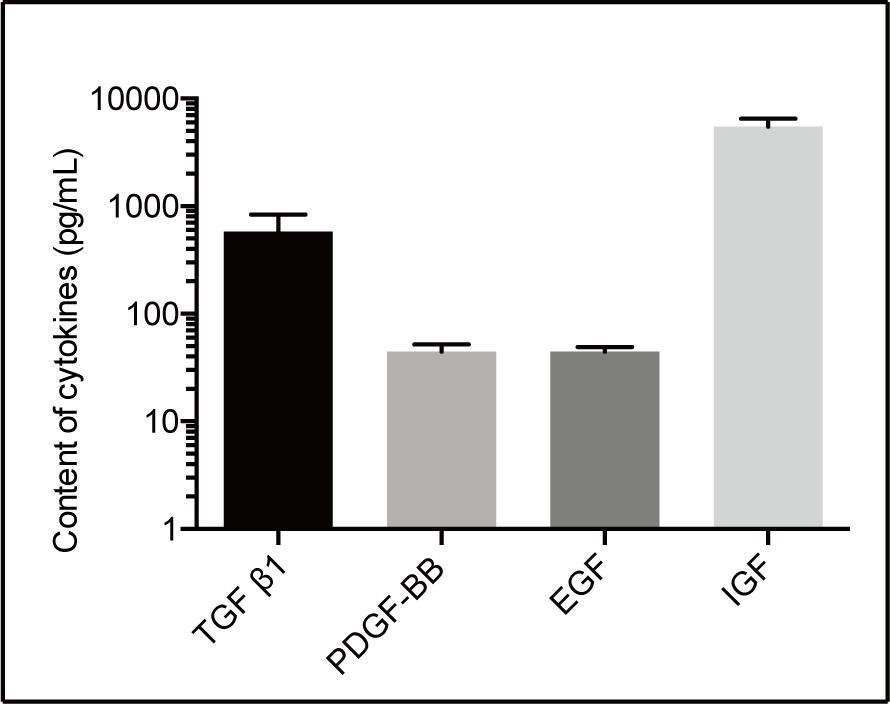

Supplement: Supplementary file 5 — Additional file 4: Figure S4. Levels of cytokines measured from HS5 culture supernatants. Various cytokines in the culture supernatants were measured by ELISAs. [file 12964_2020_592_MOESM5_ESM.tif]

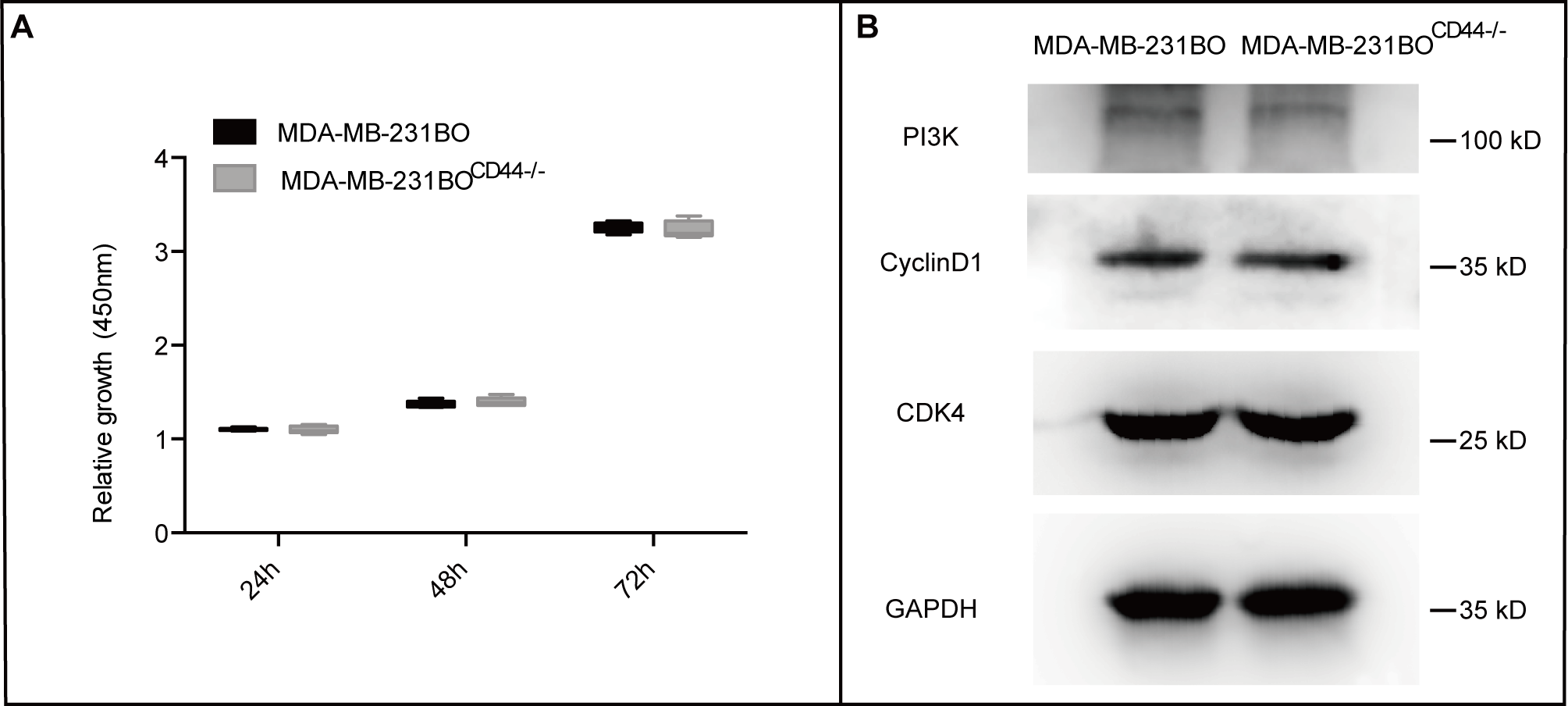

Supplement: Supplementary file 6 — Additional file 5: Figure S5. The effect of CD44 on breast cancer cell growth. A. CCK-8 proliferation assay was used to detect the growth of MDA-MB-231BO cells after downregulation of CD44. Bars represent mean ± SEM. B. Western blot was used to detect the expression of signalling proteins, including PI3K, Cyclin D1, and CDK4 after downregulation of CD44. [file 12964_2020_592_MOESM6_ESM.tif]
