# Supplementary material for: Remodelling of the bone marrow microenvironment by stromal hyaluronan modulates the malignancy of breast cancer cells
Source: Cell Commun Signal. 2020 Jun 9;18:89. doi: 10.1186/s12964-020-00592-z (PMC7285718; doi:10.1186/s12964-020-00592-z)
Supplement: Supplementary file 7 — Additional file 6: Table 1. The number of mice of osteolysis. [file 12964_2020_592_MOESM7_ESM.docx]

**Remodelling of the bone marrow microenvironment by stromal hyaluronan modulates the malignancy of breast cancer cells**

**Xiaoyan Chen^1,2,†^, Xiaoxing Shi^3,†^, Yiwen Liu^1^, Yiqing He^1^, Yan Du^1^, Guoliang Zhang^1^, Cuixia Yang^1,4,*^, and Feng Gao^1,4,*^**

**Supplementary table 1. The number of mice of osteolysis.**

|  | MDA-MB-231BO group（ number of bone destruction mice /number of total mice） | MDA-MB-231BO^CD44-/-^ group（ number of bone destruction mice /number of total mice） |
| --- | --- | --- |
| day 11 | 4/16 | 3/16 |
| day 17 | 8/16 | 4/16 |
| day 24 | 9/16 | 4/16 |
| day 31 | 11/16 | 5/16 |

The tibial destruction was observed by Micro-CT scanning at day 0, 11, 17, 24, and 31, respectively.
